# Degradation Behavior of Surface Wear Resistance of Marine Airport Rigid Pavements

**DOI:** 10.3390/ma19010054

**Published:** 2025-12-23

**Authors:** Yuming Guo, Jingxuan Zhao, Tiancong Hao, Qingya Sun

**Affiliations:** 1 College of Transportation Engineering, Tongji University, Shanghai 200092, China; gymyjssq@163.com; 2 Department of Architecture, The University of Hong Kong, Pokfulam Road, Hong Kong SAR, China; 3 College of Materials and Engineering, Beijing University of Technology, Beijing 100124, China; wendyzhaojx@126.com; 4 School of Mechanical Engineering, Nanjing University of Science and Technology, Nanjing 210094, China; 5 School of Safety Science and Engineering, Nanjing University of Science and Technology, Nanjing 210094, China; qingyasun@sina.com

**Keywords:** marine airport pavement, wear resistance degradation, salt erosion, impact loading, coupled deterioration mechanism

## Abstract

Rigid pavements in marine airports are subjected to severe surface degradation due to the combined effects of salt erosion and repeated aircraft impact loading, which significantly reduces service life and operational safety. This study investigates the degradation behavior and underlying mechanisms governing the surface wear resistance of C40 concrete under simulated marine environmental and mechanical conditions. Specimens were first subjected to repeated drop-weight impact loading, after which abrasion tests were performed to quantify surface wear resistance. Microstructural evolution and corrosion products were characterized by X-ray diffraction (XRD) and scanning electron microscopy (SEM) analyses. The results show that repeated impact loading significantly accelerates surface deterioration: after 60 abrasion cycles, cumulative mass loss increased by up to 23.6 g for specimens subjected to 80 impacts, while long-term water absorption rose by up to 7.52% due to impact-induced microcracking. In contrast, moderate salt-fog exposure initially enhanced wear resistance, as cumulative mass loss decreased from 18.1 g (unexposed) to 9.4 g after 30 cycles, attributable to pore filling by CaCO_3_ and Friedel’s salt. However, prolonged exposure (40 cycles) reversed this trend, leading to strength loss. Under combined impact of salt-fog conditions, the wear resistance deteriorated more rapidly, and the transition from strengthening to weakening occurred earlier than under salt exposure alone, indicating a coupled degradation effect. These findings clarify the coupled chemical–mechanical deterioration mechanism of marine airport pavements and provide a scientific basis for durability design and maintenance optimization.

## 1. Introduction

Airport runways are critical infrastructures whose surface wear resistance directly affects aircraft safety and operational efficiency [1,2,3,4,5,6]. For instance, Fwa [1] demonstrated that pavement skid resistance is a key parameter for safe aircraft operations and is highly sensitive to surface texture degradation. Ali et al. [2] showed that optimizing concrete mixture composition can significantly enhance the impact performance of runway pavements under repeated aircraft loading, which were validated by Kim et al. [3] and Yuan et al. [4]. To improve durability, some studies [5,6] reported that novel surface coatings can effectively enhance the wear and scaling resistance of airport concrete pavements. In marine environments, rigid pavements are simultaneously exposed to salt attack and repeated aircraft impact loads [7,8]. Such combined mechanical and chemical stresses accelerate surface deterioration, reducing the pavement’s functional and structural lifespan [9,10]. Understanding the mechanisms governing this degradation is essential for improving the durability and reliability of marine airport pavements [8,11,12,13].

In addition to surface abrasion, marine pavement concrete is also affected by material aging, moisture transport, and chemical accumulation at the exposed surface layer. Long-term environmental actions may change the pore structure, surface roughness, and local stiffness of near-surface concrete, which in turn influence tire–pavement interaction and loading transfer behavior. Recent studies [14,15] on concrete durability in aggressive environments have demonstrated that surface degradation is often governed by the interaction between environmental exposure and mechanical response, rather than by a single deterioration mechanism. Therefore, a performance-oriented evaluation approach focusing on surface functionality is particularly important for pavement systems operating under harsh service conditions.

Previous investigations have mainly focused on single factors, such as chloride or sulfate corrosion, or static and cyclic loads [16,17,18,19,20]. These studies have revealed that salt ions can penetrate concrete, react with hydration products, and alter pore structures, while heavy loads exacerbate cracking and material loss [21,22]. However, the synergistic effect of salt erosion and impact loading remains poorly understood [2,8]. In particular, how cyclic salt exposure coupled with transient aircraft impacts influences microstructural evolution and wear resistance decay has not been systematically explored.

Moreover, most existing studies [23,24] have focused on compressive strength, mass loss, or corrosion depth as primary durability indicators, whereas systematic investigation of abrasion resistance as a functional performance index for airport pavements is relatively limited. In practical runway service, surface wear directly governs skid resistance, texture depth, and operational safety; however, its evolution under combined mechanical–chemical loading has received much less attention. This gap limits the understanding of how pavement surface integrity evolves under realistic marine operating conditions. In engineering practice, the abrasion resistance of airport concrete pavements is commonly evaluated using standardized test methods (e.g., ASTM C944 [25], GB/T 16925 [26]), in which mass loss or wear depth is used as a performance index, although no unified international limit has yet been specified for marine airport pavements. Previous study [23] has shown that repeated impact loading or heavy aircraft traffic can increase abrasion-related mass loss by inducing microcracking and loosening of the surface layer. Under saline exposure, moderate salt action may temporarily reduce surface wear due to pore filling by secondary products, whereas prolonged exposure leads to accelerated abrasion associated with cracking, leaching, and salt crystallization damage [27].

To address these limitations, this study simulated the coupled salt erosion–impact environment typical of marine airports using laboratory tests combining cyclic impact and salt-spray exposure. Surface degradation was quantitatively evaluated by abrasion testing conducted after designated impact and salt-fog exposure cycles. The evolution of surface wear resistance, microstructural degradation, and corrosion products was analyzed through wear testing, XRD, SEM, and EDS characterization. The objective was to establish the degradation law of wear resistance under combined chemical-mechanical action, identify the critical factors controlling performance decay, and provide guidance for predicting pavement life and scheduling timely maintenance.

## 2. Materials and Methods

### 2.1. Materials

#### 2.1.1. Cement

The cement used in this study was a laboratory-prepared ordinary Portland cement (P.O 42.5), conforming to the requirements of GB 175-2007 [28]. Its basic physical and mechanical properties were determined prior to use. The cement exhibited an initial setting time of approximately 140 min and a final setting time of 230 min. The flexural strength reached 5.14 MPa after 3 days and 7.28 MPa after 28 days, while the corresponding compressive strengths were 20.36 MPa and 49.3 MPa, respectively. The fineness, measured by 45 µm sieve residue, was 2.13%, and the standard consistency water requirement was about 28%. These parameters indicate that the P.O 42.5 cement possesses stable hydration performance and mechanical reliability suitable for C40 concrete preparation. The cement used in this work conforms to the requirements of GB 175-2007 for P.O 42.5 ordinary Portland cement, whose chemical composition and clinker phase proportions are regulated within standardized ranges.

#### 2.1.2. Fine Aggregate

**Figure 1:**
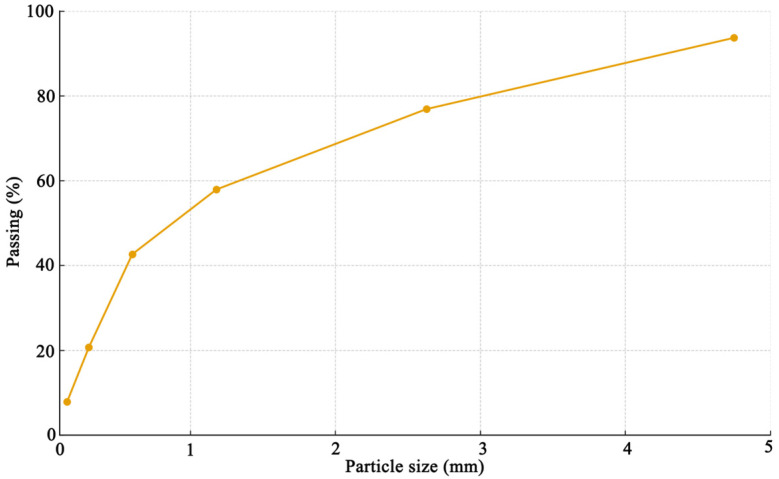
Granulometric curves of sand in this work.

Natural river sand was used as the fine aggregate in this experiment (Figure 1). The sand was air-dried before mixing and characterized according to the particle size distribution obtained from sieve analysis. The results showed a well-graded composition, with the majority of particles distributed between 0.3 mm and 2.36 mm. The cumulative sieve residue reached approximately 93%, and the fineness modulus was calculated to be 2.8, classifying it as a medium sand according to GB/T 14684-2022 [29]. The sand exhibited good cleanliness and particle roundness, ensuring adequate workability and strength development of the concrete mixture.

#### 2.1.3. C40 Concrete Specimens

The concrete used in this study was designed with a target compressive strength of C40. The water–cement ratio was set to 0.49. The mix proportion for C40 concrete was determined as follows: 449 kg/m^3^ of cement, 623 kg/m^3^ of fine aggregate (sand), 1108 kg/m^3^ of coarse aggregate (crushed stone), and 220 kg/m^3^ of water, corresponding to a water–cement ratio of 0.49. This proportion ensured adequate workability and mechanical performance for subsequent impact and salt-fog corrosion tests. No chemical admixtures were used in this study. Tap water was used for all concrete mixing and curing. The coarse aggregate used in this study was crushed stone with a nominal maximum size of 20 mm. The aggregate exhibited good cleanliness and angularity and was used in an air-dried state.

Concrete specimens were mixed in a laboratory mixer, cast into steel molds (150 mm × 150 mm × 150 mm), vibrated to remove entrapped air, and cured under standard conditions (20 ± 2 °C, RH ≥ 95%) for 28 days before testing. All experiments were repeated at least three times to ensure reliability of the results. Compressive strength tests confirmed that the mechanical properties of the commercial specimens were consistent with those of the laboratory-prepared C40 concrete, indicating negligible differences in strength and overall performance. Therefore, both sets of specimens were deemed suitable for the subsequent durability and mechanical evaluations.

### 2.2. Experimental Design

#### 2.2.1. Effect of Mechanical Impact Loading on the Wear Resistance of Concrete

**Figure 2:**
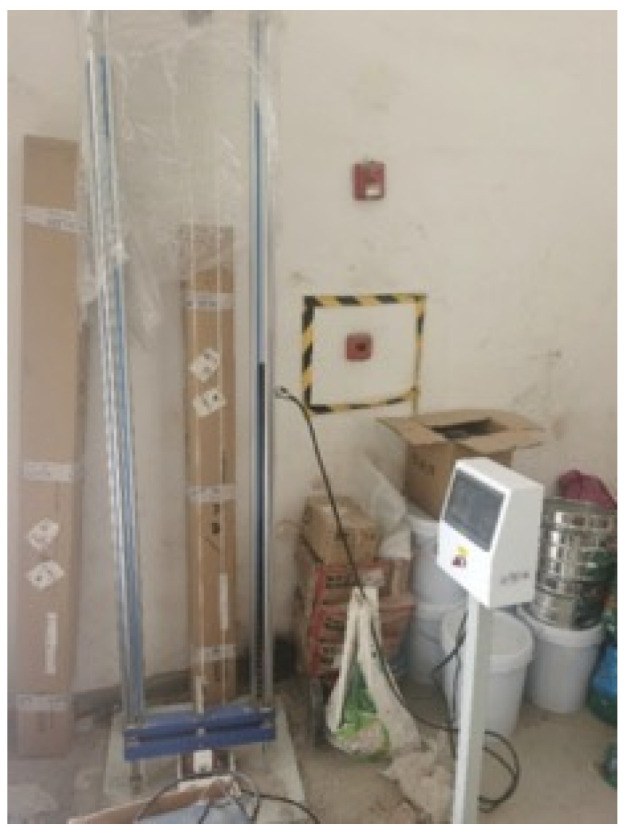
Photo for impact experiment setup.

To investigate the effect of mechanical impact loading on surface wear resistance, C40 concrete specimens were subjected to repeated drop-weight impacts using a drop hammer testing apparatus (CJ-2000, Shanghai Rongjid Experimental Instrument Co., Ltd., Shanghai, China). A steel striker (Figure 2) with a hemispherical tip was used to apply impact loads to the center of the specimen surface. The striker mass was 5 kg, and the falling height was fixed at 0.5 m. Accordingly, the initial impact velocity before contact was approximately 3.13 m·s^−1^, calculated from v = √(2gh). The specimens were impacted 0, 20, 40, 60, and 80 times, respectively. After impact loading, abrasion tests were conducted using a standard abrasion testing machine (TMS-04 concrete abrasion testing machine, Wuxi, China) to evaluate the surface wear resistance degradation caused by cumulative impacts. The corresponding specimens were denoted as I-0, I-20, I-40, I-60, and I-80, where the number indicates the number of drop-weight impacts applied. In addition, water absorption tests were performed on the impacted specimens to assess surface microstructural damage and permeability changes under different impact cycles. The specimens were fully immersed in distilled water at a constant temperature of 20 ± 2 °C for 24 h. After the immersion period, the specimens were removed, and the change in mass was determined by weighing the specimens using a digital balance with an accuracy of 0.01 g.

#### 2.2.2. Effect of Salt Fog Corrosion on the Wear Resistance of Concrete

To examine the chemical degradation process, C40 concrete blocks were exposed to cyclic salt fog corrosion using a controlled salt-spray chamber (Shanghai Changken Testing Equipment (Group) Co., Ltd., Shanghai, China). The test involved alternating wet–dry salt exposure cycles to simulate marine atmospheric conditions. The salt (3.5 wt.% NaCl solution)-fog corrosion tests were conducted using a programmable salt-spray chamber equipped with two independent spraying systems: a salt-fog spraying system and a simulated rainfall (shower) spraying system. The test protocol was designed with reference to GB/T 10125 [30] and ISO 9227 [31], but modified to include alternating rainfall and drying phases to better simulate marine atmospheric exposure conditions. Both spraying modes allow adjustable spray volume and nozzle angle to ensure uniform exposure of the specimen surface. The chamber is also equipped with two separate solution tanks, including a pure-water tank and a salt-solution tank, enabling automatic switching between solutions during programmed cycles. A complete wet–dry corrosion cycle lasted 24 h and consisted of four sequential stages: 4 h drying phase, 8 h salt-fog spraying, 4 h drying phase, and 8 h freshwater spraying. The cycle sequence was automatically controlled by the internal program to simulate marine atmospheric exposure conditions including salt deposition, rainfall, and drying. The specimens were labeled as S-0, S-10, S-20, S-30, and S-40, corresponding to 0, 10, 20, 30, and 40 salt fog cycles, respectively. After each cycle group, the surface abrasion resistance was measured to determine the influence of salt-induced corrosion on surface wear behavior. Furthermore, selected samples were characterized by X-ray diffraction (XRD) and other microstructural analysis techniques to identify corrosion products, assess chemical alteration, and observe morphological evolution within the concrete matrix. For XRD analysis, material was collected from the surface layer of the specimens after the exposure cycle. XRD analysis was carried out using a diffractometer (X’Pert PRO MPD, Malvern Panalytical Ltd., Almelo, The Netherlands) equipped with Cu Kα radiation (λ = 1.5406 Å). The operating voltage and current were set to 40 kV and 40 mA, respectively. Data were collected over a 2θ range from 10° to 60°, with a step size of 0.02° and a scanning rate of 2°·min^−1^. Prior to testing, the concrete surface layer was carefully scraped, ground into fine powder, and dried at 60 °C to remove moisture. Phase identification was performed by comparison with the ICDD PDF-4+ database.

#### 2.2.3. Combined Effect of Drop-Weight Impact and Salt Fog Corrosion on the Wear Resistance of Concrete

To simulate the coupled mechanical and chemical deterioration encountered in marine airport pavements, a combined drop-impact and salt-fog corrosion experiment was designed. Each combined cycle consisted of an initial mechanical loading stage, during which the C40 concrete specimen was subjected to 20 drop-weight impacts, followed by 10 wet–dry salt fog cycles in the salt-spray chamber. After the prescribed number of combined cycles, abrasion tests were performed to quantify the synergistic effect on surface wear resistance. Specimens were denoted as IS-0-0, IS-20-10, IS-40-20, IS-60-30, and IS-80-40, where the first number represents the total number of impact loadings and the second number corresponds to the number of salt fog cycles. This design enables systematic evaluation of the interactive influence of impact damage and salt corrosion on the microstructural integrity and wear durability of concrete.

## 3. Results and Discussion

### 3.1. Effect of Repeated Impact Loading on the Wear Resistance of Concrete

**Figure 3:**
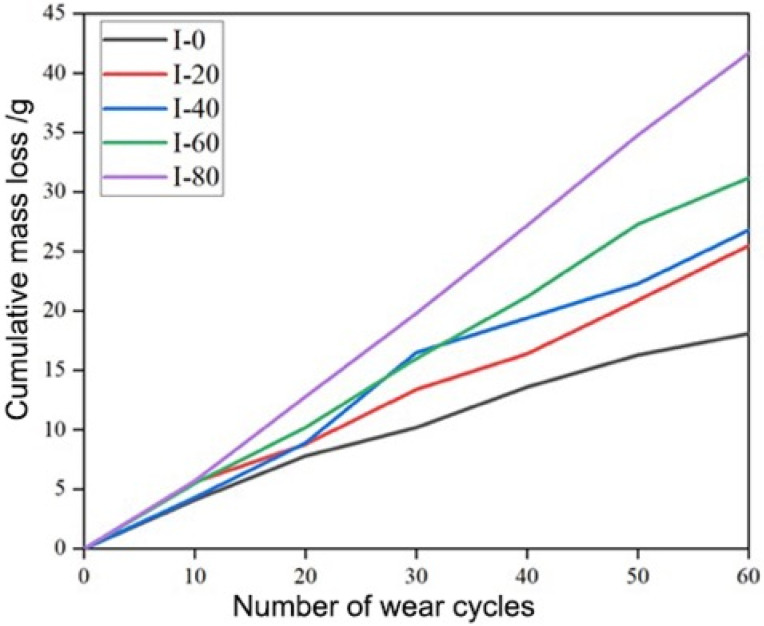
Cumulative mass loss of C40 concrete specimens measured by abrasion testing after repeated impact loading under different numbers of impact cycles (I-0, I-20, I-40, I-60, and I-80).

**Figure 4:**
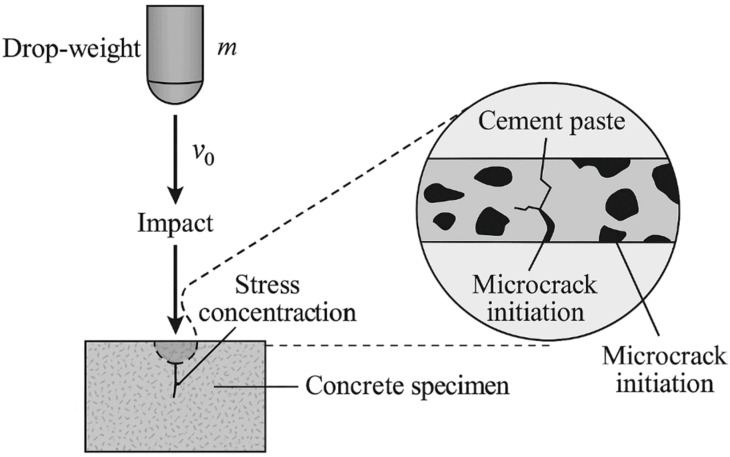
The diagram for illustrating the stress concentration.

**Figure 5:**
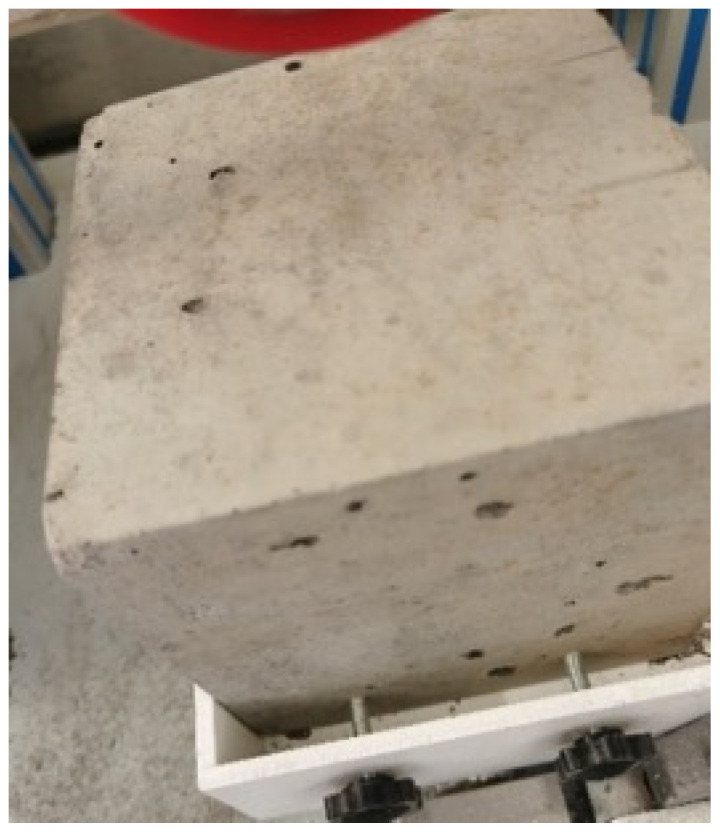
Image showing specimens after impact loading.

The variation in cumulative mass loss of C40 concrete specimens under repeated drop-weight impacts is presented in Figure 3. Five sets of specimens were subjected to 0, 20, 40, 60, and 80 impact cycles, denoted as I-0, I-20, I-40, I-60, and I-80, respectively. After impact treatment, surface abrasion tests were performed using a standardized abrasive (silicon carbide), with 1000 abrasion revolutions per cycle and a 5 min duration per cycle. The specimen mass loss was recorded at intervals of every ten abrasion cycles. As shown in Figure 3, all specimens exhibited a linear increase in cumulative mass loss with increasing abrasion cycles, indicating a gradual deterioration of the surface layer. However, the slope of the mass–loss curve increased significantly with the number of prior impact loads. Compared with the unimpacted specimen (I-0), the total mass loss after 60 abrasion cycles increased by approximately 7.4 g, 8.7 g, 13.1 g, and 23.6 g for the I-20, I-40, I-60, and I-80 specimens, respectively. This progressive rise in abrasion loss demonstrates that repeated impact loading induces micro-cracking and structural weakening within the concrete surface layer [32,33]. The cumulative mechanical energy input from impact loading likely generates localized tensile stresses (Figure 4 and Figure 5), which promote the initiation and propagation of micro-cracks at the cement paste–aggregate interface. These micro-defects reduce the surface compactness and increase the susceptibility of mortar and aggregate particles to detachment during abrasion. Consequently, the wear resistance of the concrete surface decreases markedly with increasing impact cycles [34].

**Figure 6:**
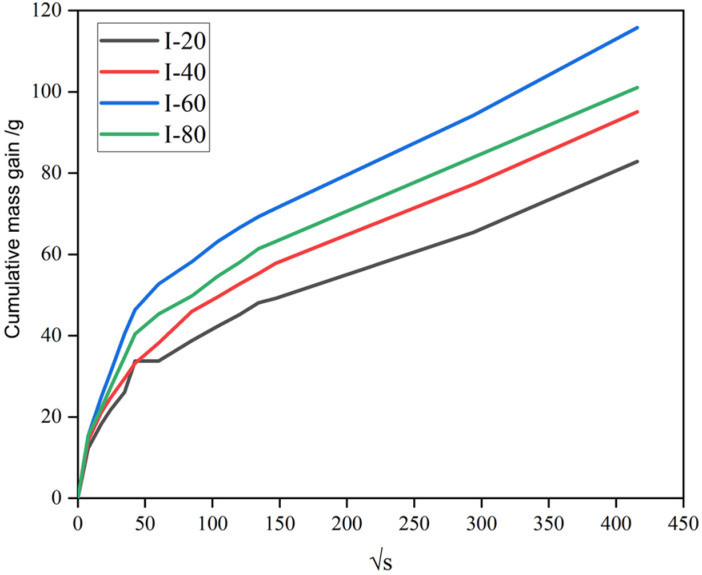
Cumulative water absorption plotted against the square root of time for C40 concrete specimens after impact loading, for different numbers of impact cycles (I-20, I-40, I-60, and I-80).

To evaluate the influence of repeated impact loading on the internal pore structure and permeability of concrete, unidirectional water absorption tests were conducted. Four C40 concrete specimens that had previously undergone 20, 40, 60, and 80 drop-weight impacts were selected and labeled as I-20, I-40, I-60, and I-80, respectively. Prior to testing, all specimens were oven-dried at 60 °C for 24 h to a constant mass and then sealed with waterproof adhesive tape on four lateral faces and the bottom surface, ensuring that only the top face was exposed to water. Each specimen was weighed and then placed upside-down in a water container, resting on metal spacers to prevent direct contact between the concrete surface and the container bottom. The water level was maintained at 2 ± 1 mm above the exposed surface, and the test began immediately upon first contact between water and the specimen surface. The mass gain of each specimen was recorded at 13 time intervals: 1 min, 5 min, 10 min, 20 min, 30 min, 1 h, 2 h, 3 h, 4 h, 5 h, 6 h, 24 h, and 48 h. The first measurement was taken at 60 ± 2 s, the second at 5 ± 10 s, the next four points within ±2 min, and the 24 h and 48 h readings within ±1 min of the scheduled time. The experimental results are shown in Figure 6, which presents the relationship between cumulative mass increase and the square root of time. The mass gain curves exhibited a two-stage behavior: a rapid increase in the initial absorption stage (within approximately the first 50 s^1^ᐟ^2^), followed by a slower, quasi-linear growth phase. Moreover, the cumulative water uptake of the specimens increased significantly with the number of prior impact loadings. After 48 h of exposure, the total absorbed water mass of I-80 was nearly twice that of I-20, indicating that repeated impact loading substantially enhanced capillary suction.

This phenomenon can be attributed to the formation of micro-cracks and micro-voids within the surface layer and interfacial transition zones as a result of cyclic impact energy. These micro-defects create preferential flow paths that facilitate water ingress, thereby increasing the apparent sorptivity of the material. The increase in cumulative absorption with higher impact cycles confirms that repeated mechanical loading leads to progressive deterioration of the concrete’s internal compactness and a reduction in its resistance to moisture penetration.

**Figure 7:**
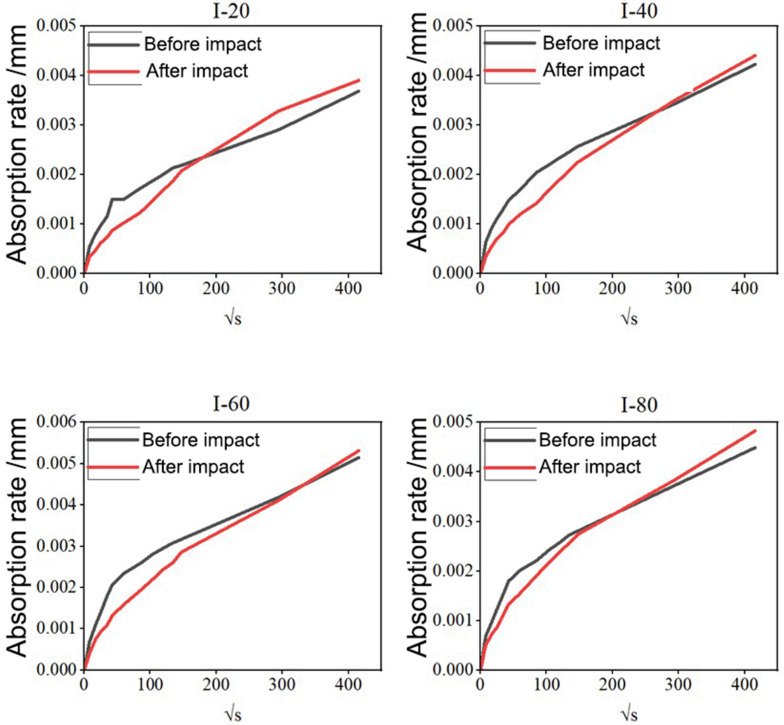
Comparison of water absorption of C40 concrete specimens before impact loading (black) and after repeated impact loading (red) under different numbers of impact cycles (I-20, I-40, I-60, and I-80). The absorption rate was measured using a standardized method, with measurements taken at regular intervals using a high-precision digital caliper and a balance with 0.001 g accuracy.

Figure 7 illustrates the variation in water absorption rate before and after impact loading for concrete specimens subjected to different numbers of drop-weight impacts (I-20, I-40, I-60, and I-80). In each subfigure, the black curve represents the pre-impact specimen, while the red curve corresponds to the same specimen tested after impact treatment. As shown in Figure 7, during the early stage of water absorption (within approximately 1 h), all specimens exhibited a rapid increase in sorptivity, followed by a gradual reduction in the absorption rate. After about 1 h, the water uptake rate of the pre-impact specimens began to slow markedly, while that of the post-impact specimens remained relatively steady. As a result, the two curves progressively converged, and after 24 h, the cumulative water absorption of the post-impact specimens surpassed that of the pre-impact ones. Quantitatively, the overall water absorption increased by 5.91%, 4.21%, 3.28%, and 7.52% for specimens I-20, I-40, I-60, and I-80, respectively. These results indicate that although impact loading initially reduces the surface sorptivity due to compaction or surface densification, the repeated mechanical impacts simultaneously generate micro-cracks and loosen the internal matrix. The resulting increase in pore connectivity and micro-void volume enhances capillary suction within the bulk concrete. Consequently, at later stages, the total water absorption of impacted specimens becomes higher than that of unimpacted specimens, reflecting a loss of internal compactness and an increased susceptibility to moisture ingress.

### 3.2. Effect of Salt-Fog Exposure on the Wear Resistance of Concrete

To investigate the effect of salt-fog corrosion on the abrasion resistance of concrete, C40 specimens were exposed to controlled salt-fog environments for different numbers of wet–dry cycles. The specimens were labeled S-0, S-10, S-20, S-30, and S-40, corresponding to 0, 10, 20, 30, and 40 salt-fog cycles, respectively. During testing, all samples were placed on a stainless-steel rack inside a salt-fog chamber, ensuring sufficient spacing between specimens for uniform exposure. The chamber’s distilled-water and reagent tanks were filled with pure water and artificially simulated seawater, respectively. Additional water was added to the channel on the chamber lid to maintain a sealed and humid environment. Continuous water replenishment was ensured through an auxiliary pipe to keep the internal system hydrated throughout the experiment.

**Figure 8:**
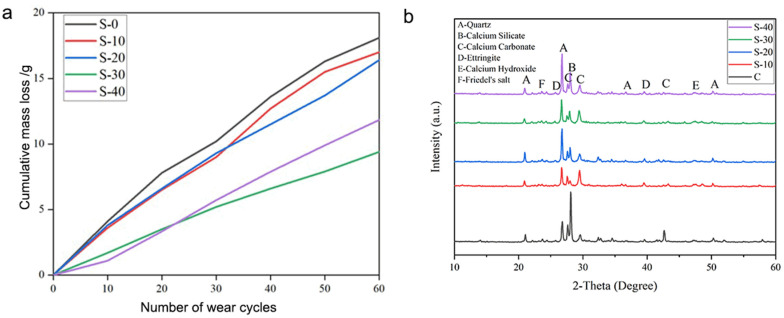
(**a**) Cumulative mass loss of C40 concrete specimens subjected to different numbers of salt-fog wet–dry cycles (S-0, S-10, S-20, S-30, S-40). (**b**) XRD spectrum for the identification of crystalline phases of concretes after salt-fog corrosion.

After the designated number of salt-fog cycles, the specimens were removed and subjected to surface abrasion testing. The cumulative mass loss was recorded every ten abrasion cycles to quantify the effect of salt-fog exposure on surface wear resistance. The test results are shown in Figure 8. The cumulative mass loss of the specimens decreased with increasing numbers of salt-fog cycles up to 30 cycles, indicating an improvement in wear resistance. After 60 abrasion cycles, the total mass loss values were approximately 18.1 g, 17.0 g, 16.4 g, and 9.4 g for S-0, S-10, S-20, and S-30, respectively. This reduction in material loss suggests that the salt-fog environment initially promoted surface densification through continued hydration and salt crystallization within surface pores, leading to enhanced abrasion resistance [10,17]. However, after 40 wet–dry cycles, the cumulative mass loss increased to 11.8 g, revealing a decline in surface durability. This reversal indicates that prolonged salt-fog exposure eventually caused microcrack propagation, increased pore connectivity, and surface scaling, which outweighed the initial strengthening effect.

Powder samples were collected from the surface layers of the S-10, S-20, and S-30 specimens during the abrasion tests and analyzed using XRD to identify the crystalline phases formed after salt-fog corrosion. The XRD patterns are presented in Figure 8b. The major crystalline phases detected in the concrete surfaces after salt-fog exposure may include calcium carbonate (CaCO_3_) [35], Friedel’s salt (Ca_2_Al(OH)_6_Cl·10H_2_O), and ettringite (AFt, Ca_6_Al_2_(SO_4_)_3_(OH)_12_·26H_2_O), along with peaks corresponding to quartz (SiO_2_), calcium silicate, and calcium hydroxide (Ca(OH)_2_). However, no definitive phase identification is claimed in the absence of quantitative analysis and complementary chemical characterization. The appearance of Friedel’s salt and AFt reveals that chloride ions in the artificial seawater solution reacted with the hydration products of cement, leading to the formation of chloride-bearing and sulfate-bearing compounds. This demonstrates that the salt-fog environment effectively initiated chemical interactions between saline ions and the cementitious matrix. However, no diffraction peaks corresponding to expansive products such as gypsum (CaSO_4_·2H_2_O), magnesium hydroxide (Mg(OH)_2_), magnesium silicate hydrate (M-S-H), or thaumasite (TSA) were observed. This suggests that the duration of salt-fog exposure was insufficient to promote the formation of expansive crystalline salts, and thus no significant structural disruption occurred within the concrete matrix. Moreover, the presence of newly formed CaCO_3_ indicates a secondary carbonation process in which carbon dioxide dissolved in the salt-fog solution reacted with calcium hydroxide to form calcite. The precipitation of CaCO_3_ within surface pores contributed to partial pore filling and surface densification, thereby improving the abrasion resistance of the specimens after moderate salt-fog cycling.

**Figure 9:**
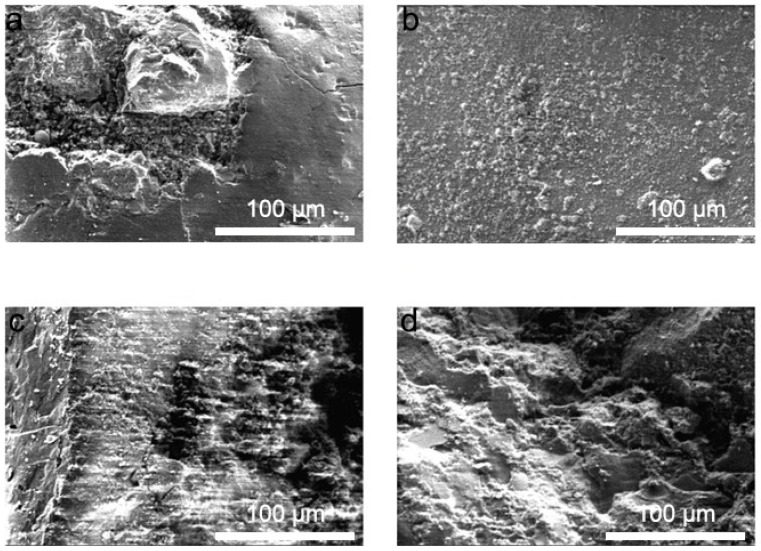
SEM micrographs of concrete specimens: (**a**) surface of control specimen C-7; (**b**) surface of S-40 after 40 salt-fog cycles; (**c**) cross-section of S-10; and (**d**) cross-section of S-40.

The SEM observations (Figure 9) revealed that salt-fog exposure caused noticeable surface densification with crystalline deposits in the outer layer, while microcracks and salt crystals were mainly confined to the near-surface zone and did not penetrate deeply into the concrete matrix. It should be noted that EDS analysis was not conducted in the present study. Therefore, SEM observations were used solely for morphological interpretation, and phase identification was based exclusively on XRD results.

### 3.3. Effect of Combined Impact Loading and Salt-Fog Cycles on the Wear Resistance of Concrete

**Figure 10:**
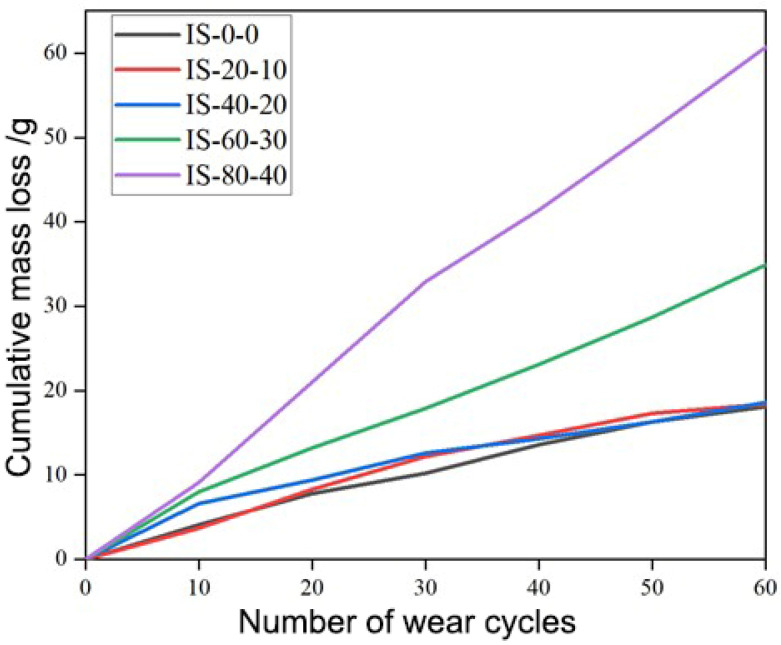
Cumulative mass loss of C40 concrete specimens under different combined impact–salt-fog cycles (IS-0-0, IS-20-10, IS-40-20, IS-60-30, and IS-80-40).

To evaluate the coupled mechanical–chemical degradation of concrete under service-like marine conditions, a combined impact–salt-fog cycling test was designed using C40 concrete specimens. One combined cycle consisted of 20 drop-weight impacts followed by 10 salt-fog wet–dry cycles. Five specimens were prepared and subjected to 0, 1, 2, 3, and 4 combined cycles, respectively, and were labeled IS-0-0, IS-20-10, IS-40-20, IS-60-30, and IS-80-40. After treatment, abrasion tests were performed to determine the cumulative mass loss of each specimen surface, as shown in Figure 10. Moderate combined cycling initially exerted a compensating effect between the two processes. After 20 impact–10 salt-fog cycles and 40 impact–20 salt-fog cycles, the total mass loss after 60 abrasion cycles showed little difference compared with the untreated specimen, suggesting that the beneficial densification effect of salt-fog exposure counterbalanced the micro-damage induced by impact loading. However, when the number of combined cycles increased to 60 impact–30 salt-fog and 80 impact–40 salt-fog, the cumulative mass loss rose sharply, indicating severe deterioration of surface integrity. The wear resistance of concrete decreased markedly under high-frequency impact–corrosion coupling due to progressive cracking, pore coarsening, and loss of surface cohesion.

**Figure 11:**
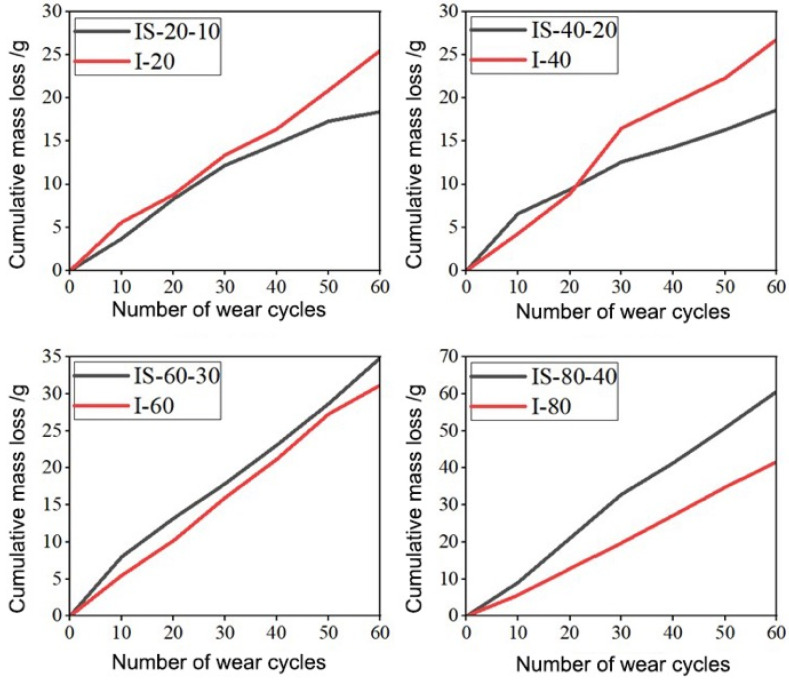
Comparison of cumulative mass loss between impact-only (I-series) and combined impact–salt-fog (IS-series) specimens.

Figure 11 compares the cumulative mass loss of specimens subjected solely to impact loading (I-series) with those exposed to combined impact–salt-fog cycles (IS-series). Specifically, each IS specimen (IS-20-10, IS-40-20, IS-60-30, and IS-80-40) corresponds to its impact-only counterpart (I-20, I-40, I-60, and I-80), allowing direct evaluation of the salt-fog effect on surface wear behavior. As shown in Figure 11, the specimens IS-20-10 and IS-40-20 exhibited lower cumulative mass losses than their corresponding impact-only specimens (I-20 and I-40), indicating that moderate salt-fog exposure enhanced the wear resistance of the concrete surface. This improvement can be attributed to the partial pore filling and surface densification caused by the formation of calcium carbonate and other crystallization products during the early stages of salt-fog corrosion. In contrast, when the number of combined cycles reached IS-60-30 and IS-80-40, the mass loss became significantly greater than that of I-60 and I-80, respectively. This reversal trend suggests that after approximately 30 impact–salt-fog cycles, the reinforcing effect of salt crystallization was replaced by a deterioration mechanism dominated by microcracking, ion ingress, and matrix softening. The transition from strengthening to weakening occurred earlier under combined loading than under salt-fog exposure alone, implying that impact loading accelerates the onset of corrosion-induced damage. From a practical perspective, this finding suggests that the optimal maintenance interval for airport concrete pavements in marine environments should be determined based on the transition point at which salt-fog exposure changes from beneficial densification to detrimental degradation. Timely surface maintenance at this critical stage could effectively delay the deterioration of wear resistance under service conditions. It should be noted that no quantitative XRD analysis, SEM/EDS verification, or cement chemical characterization was performed in the present study. Therefore, chemical processes such as Friedel’s salt formation, carbonation, or chloride binding were not experimentally confirmed and are discussed only in the context of reported mechanisms in the literature.

**Figure 12:**
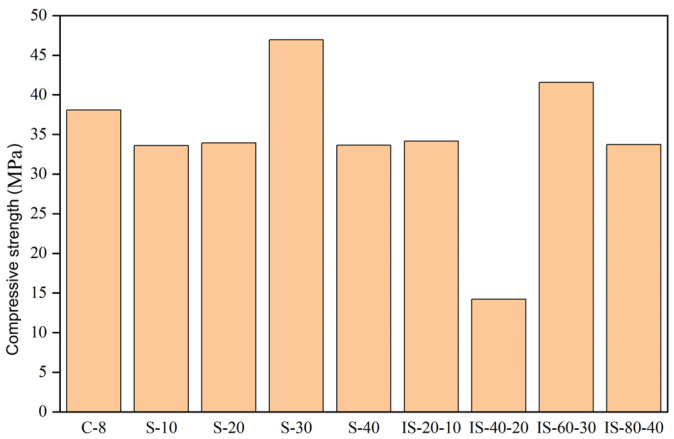
The 28-day compressive strength of C40 concrete specimens under different exposure conditions, including control (C-8), salt fog cycles (S-10, S-20, S-30, S-40), and combined impact–salt fog cycles (IS-20-10, IS-40-20, IS-60-30, IS-80-40).

The compressive strength of the C40 concrete was measured at 28 days. Figure 12 shows the reference specimens (C-8). Under salt-fog exposure alone, specimens subjected to 10–30 cycles exhibited an increase in compressive strength, reaching a maximum at S-30, followed by a decrease at S-40. Under combined impact–salt conditions, the compressive strength decreased significantly, with IS-40-20 showing a pronounced strength reduction. These results indicate that moderate salt exposure can temporarily enhance concrete strength, whereas combined mechanical and environmental loading leads to accelerated strength degradation.

**Figure 13:**
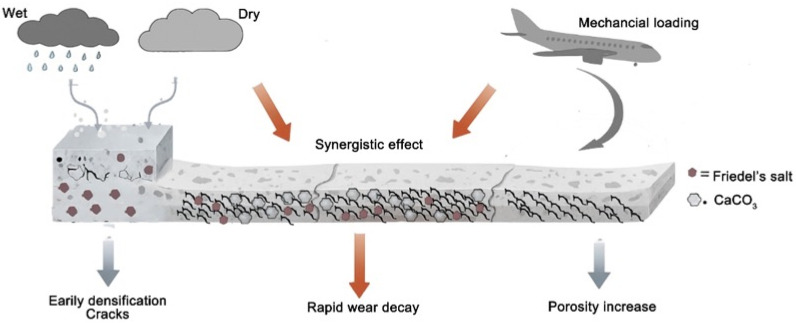
Schematic mechanism of wear-resistance evolution of airport concrete under coupled salt-fog wet–dry cycling and repeated mechanical loading.

Figure 13 presents the schematic mechanism of wear-resistance evolution of airport concrete under coupled salt-fog wet–dry cycling and repeated mechanical loading. During the early stage of salt-fog exposure (wet–dry cycles), chloride-bearing solution reacts with surface hydration products to form Friedel’s salt and secondary CaCO_3_ (via carbonation of Ca(OH)_2_), which partially fills pores and densifies the outer layer; this yields a transient improvement in abrasion resistance (consistent with S-10/S-20/S-30). Repeated mechanical impacts introduce tensile stresses that initiate and propagate microcracks in the surface mortar and interfacial transition zones. Under combined action, the densified skin is progressively fractured; cracks open and interconnect with pre-existing pores, increasing effective porosity and capillary pathways (as indicated by higher long-term water uptake and the IS-series results). The coupled effect between impact-induced microcracking and salt ingress accelerates surface material loss, shifting the strengthening/weakening transition to earlier cycles than salt exposure alone (IS-60-30/IS-80-40 vs. S-40). Overall, the figure highlights: (i) early densification by CaCO_3_/Friedel’s salt near the surface, (ii) microcrack accumulation under impacts, and (iii) a synergistic deterioration phase where porosity rises and rapid wear decay ensues.

## 4. Discussion

The present results highlight how mechanical impact loading and marine-like salt fog exposure jointly control the surface wear degradation of C40 concrete. Repeated drop-weight impacts alone led to a monotonic increase in abrasion mass loss and long-term water absorption, indicating progressive microcracking and loss of surface integrity. Similar trends have been reported when concrete is pre-damaged by mechanical loading and then exposed to aggressive environments such as freeze–thaw or salt solutions, where initial load damage increases permeability and accelerates stiffness and strength loss [5,23,36,37]. In our tests, the combined impact–salt cycles did not produce a strictly super-additive (“synergistic”) effect in the mathematical sense, but they did shift the onset of rapid wear degradation to earlier cycles compared with salt exposure alone. This behavior is consistent with previous observations that coupled environmental–mechanical actions, rather than single factors, control the long-term durability of pavement concrete and marine concretes [38,39,40,41,42].

The salt-fog-only series further shows a characteristic transition from initial densification to subsequent degradation. Moderate numbers of wet–dry salt cycles reduced abrasion mass loss, which can be attributed to pore filling and surface densification by secondary products such as CaCO_3_ and chloride-bearing phases, as widely observed in concretes subjected to repeated wet–dry cycles in marine or sulfate solutions [43,44,45,46]. However, prolonged cycling led to increased wear and microstructural damage, in line with studies showing that extended wet–dry or freeze–thaw exposure in saline environments ultimately promotes cracking, coarsening of the pore structure, and loss of mechanical performance. Compared with previous work focused on strength or mass loss under coupled actions [47,48], this study specifically quantifies the evolution of surface abrasion resistance of airport-grade concrete under idealized impact–salt sequences, complementing recent research on pavement abrasion, surface treatments and coatings for airport concrete. These findings suggest that maintenance and protection strategies for marine airport pavements should not only aim at improving intrinsic abrasion resistance, but also at mitigating the detrimental interaction between repeated impact loading and cyclic salt exposure.

## 5. Conclusions

This study provides a systematic investigation of the surface wear degradation of C40 concrete under repeated impact loading, salt-fog wet–dry cycling, and their combined action, offering new insight into the coupled chemical–mechanical deterioration mechanism relevant to marine airport pavements. Quantitatively, repeated impact loading significantly accelerated surface damage: after 60 abrasion cycles, cumulative mass loss increased by up to 23.6 g for specimens subjected to 80 impacts, while long-term water absorption increased by as much as 7.52%, indicating progressive microcracking and enhanced permeability. In contrast, moderate salt-fog exposure alone temporarily improved abrasion resistance, as mass loss decreased from 18.1 g (S-0) to 9.4 g (S-30) due to pore filling by corrosion products such as Friedel’s salt and CaCO_3_. However, prolonged exposure (40 cycles) reversed this trend, leading to structural deterioration and increased surface damage.

Under combined impact–salt-fog conditions, the transition from salt-induced strengthening to wear-dominated degradation occurred significantly earlier than under salt exposure alone, demonstrating that mechanical damage accelerates the degradation pathway rather than simply superimposing on chemical attack. The novelty of this work lies in identifying and quantifying this transition behavior and revealing that impact loading shifts the durability regime of marine concrete from a temporary densification stage to an accelerated wear stage. These findings provide a mechanistic basis for durability-based maintenance planning and suggest that preventive intervention should be scheduled before the critical transition point to effectively extend the service life of marine airport pavements. Future research could focus on further exploring alternative materials and more realistic environmental conditions to enhance the durability of concrete under combined mechanical and chemical stresses.

## Data Availability

The original contributions presented in this study are included in the article. Further inquiries can be directed to the corresponding author.
